# Orthographic Neighbourhood Size Effects in Chinese Character Recognition: Small, Inconsistent, and Theoretically Ambiguous

**DOI:** 10.5334/joc.505

**Published:** 2026-06-19

**Authors:** Yixia Wang, Peter Hendrix, Emmanuel Keuleers

**Affiliations:** 1 Department of Computational Cognitive Science, Tilburg University, Warandelaan 2, 5037 AB Tilburg, Netherlands

**Keywords:** orthographic neighbourhood size, Chinese character similarity, visual word recognition, lexical decision

## Abstract

Existing orthographic neighbourhood size measures for Chinese words rely on the representation of words or characters as consisting of two components, which leads to coarse measurements. Recently, Wang and Keuleers (2024) proposed the use of Ideographic Description Sequences (IDSs) which encode the basic stroke patterns of characters and their spatial relationships to create more precise neighbourhood measures. In two studies, we compared the effect of different neighbourhood size measures on recognition of single Chinese characters, while controlling for character frequency and number of strokes. In Study 1, we replicated the conventional two-unit approach using neighbourhood size measures for semantic (N-sc) and phonetic (N-pc) components. We found no effect of N-pc or N-sc. In Study 2, we used IDS-based measures to represent orthographic similarity at a more detailed level. In Study 2a, we found that the effect of a neighbourhood size measure based on single-component substitution (N-ch) was inhibitory but weak. In Study 2b, we found that a neighbourhood density measure using weighted edit distance (WLD-10) showed facilitation. In Study 2c, we found that this facilitatory effect was retained with a normalised measure (WND-10). While conceptually the IDS-based measures are better neighbourhood measures and one might hope that this would also imply stronger neighbourhood effects, our results show that the effects of neighbourhood size on character recognition remained small and difficult to interpret theoretically. Our results suggest that if any meaningful effects of orthographic neighbourhood size exist for character recognition in Chinese, they are probably small and inconsistent.

## Introduction

Among the taxonomies proposed to categorise writing systems (Sproat and Gutkin, 2021), DeFrancis (2022) broadly distinguishes among consonantal, alphabetic, morphosyllabic, and pure syllabic writing systems. Research on visual word recognition has mostly focused on languages with alphabetic systems—where words are encoded as linear sequences of letters corresponding to speech segments (Perfetti and Sandak, 2001). However, there is also a substantial amount of work on other scripts, such as Chinese (e.g., Taft and Zhu, 1997; Tsai et al., 2006) and Hebrew (e.g., Velan and Frost, 2007; Stein et al., 2024). An important question in this regard is to what extent the same principles underlie visual word recognition across scripts.

Previous work in visual word recognition has centred on looking at effects of a range of variables across scripts, such as word frequency, word length, age of acquisition, imageability, number of strokes, concreteness, semantic transparency and so on (e.g., Stein et al., 2024; Su et al., 2023; Sze et al., 2014). Most of the these variables have an equivalent interpretation across scripts. For the *word frequency effect*, for instance, we can understand the effect as follows: as the frequency of the word increases in the environment, that word tends to be recognised faster (Brysbaert et al., 2018). Similarly, for the *age of acquisition effect*, we understand that early-acquired words are easier to process, possibly because first-learned instances are often among the most common examples of a certain semantic category (Brysbaert and Ellis, 2016).

However, the mechanisms underlying the effects of variables taking the orthographic form as input are likely to be script-dependent. For example, *word length* is a straightforward measure of counting of the number of characters, i.e., letters in alphabetic scripts (Keuleers et al., 2012). In Chinese script, however, word length is a constrained measure, as most words consist of one or two characters. A more relevant concept is *number of strokes* (NStrokes), which measures the number of brush strokes in writing a character. Another instance is the *semantic transparency effect*. In alphabetic languages such as Dutch (Zwitserlood, 1994) and English (Frisson et al., 2008), this refers to the semantic relationship between compound words and their constituent words, while in Chinese this often applies to characters and their semantic components (e.g., Su et al., 2023).

Importantly, however, these variables have been often treated as if they have an equivalent interpretation. In this context, the current paper concerns the *effect of orthographic neighbourhood size* (NS), which is often interpreted in the same way across scripts: orthographic similarity between words affects word recognition. We will discuss how the operationalisation of orthographic neighbourhood size can vary substantially in Chinese and investigate how these different operationalisations affect the interpretation of orthographic neighbourhood size in Chinese character recognition. In what follows, we will start by introducing the characteristics of the Chinese writing system that are relevant to the form-based measures. Then, we will contrast the operationalisation and empirical findings of neighbourhood size in alphabetic scripts and in Chinese. Finally, we will present a series of studies in which we relate different neighbourhood size measures in Chinese to lexical decision performance.

### The Chinese writing system

At least three features of the Chinese writing system are relevant in connection to orthographic neighbourhood measures. First, Chinese has a large set of characters, around 8,000 (*List of Commonly Used Standard Characters*; Language and Text Information Management Department, 2013), among which 3,500 are frequently used. These characters are mostly words themselves and combine to form words of up to four characters. In contrast, alphabetic languages’ vocabularies are expressed in writing using a much smaller number of letters (e.g., 26 letters in English). A written word, in this case, will often resemble many other words closely, reflecting the fact that many words in natural languages have a high degree of phonological similarity. This makes the concept of orthographic neighbourhood evident and easily interpretable for alphabetic scripts. In Chinese, the concept is less straightforward: due to the vast character inventory, on average, the orthographic overlap of words is much lower.

Second, the Chinese script is morphosyllabic, meaning each character has its own pronunciation(s). As a result, there is no single unit in Chinese that maps onto the concept of *letter*. This implies that there is no single clear unit over which orthographic neighbourhood size should be calculated.

**Figure 1:**
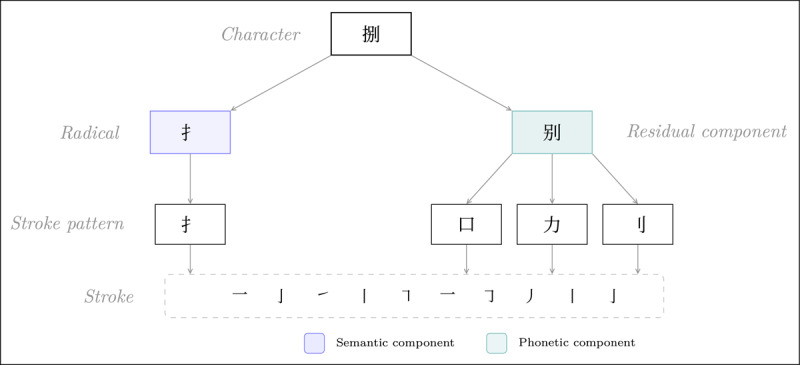
An example character 捌 *ba1* ‘eight’ to illustrate the hierarchical structure of Chinese characters. It denotes the number ‘eight’ in a formal context or refers to a specific agricultural tool. The components of characters can be categorised at different levels. Strokes form stroke patterns, which assemble into a radical and a residual component (i.e., the non-radical part), and further form the whole structure. In addition, for many characters their components at the radical levels usually manifest linguistic features. The radical 扌 serves as a semantic component meaning ‘hand’, while the residual component 别 *bie2* serves as a phonetic component, sharing its onset consonant /b/ with the character’s pronunciation *ba1*.

Third, Chinese characters are **hierarchical**. In its composition, a character can be decomposed into components at different levels of granularity: radicals, stroke patterns, or strokes.^1^ See Table 1 for definitions and Figure 1 for an example character. At each level, components can be arranged in spatial relationships that are often complex. For example, at the radical level, components can be positioned such that one semi-encloses the other, such as 凵 and 㐅 of the character 凶 *xiong1* ‘unfortunate’. At the stroke pattern level, the spatial arrangements are even more intricate, due to finer-grained components involved, such as 口, 力, and 刂, of the character 别*bie2* ‘farewell’. At the most basic level, strokes within a single character can remain parallel (e.g., 二), connect (i.e., 丁), or intersect (e.g., 十). This spatial dynamics of strokes is clearly manifested in, for instance, character 于 *yu2* ‘a common surname’. In contrast, for alphabetic languages, the spatial relation between letters or letter clusters is much simpler; it is **linear**. This makes the computation of string distances straightforward in alphabetic scripts but not in Chinese.

**Table 1:** Sub-character units. In the literature the term *radical* is used flexibly to refer to various parts of a character: the indexing component, the residual component, a general stroke pattern, among others; we adopt the first definition. The term 部件 is translated as ‘Chinese character component’ in the GF 0014-2009 standard (National Language Commission, 2009). We instead use *stroke pattern* to differentiate it from the broader use of *component* elsewhere in the paper, and to emphasise its close link to strokes. Stroke patterns vary in stroke complexity, ranging from single-stroke components (e.g., 一 in character 丛) to compound components composed of multiple stroke patterns (e.g., 相 in character 想).


TERM	DEFINITION

Radical 部首 *bu4 shou3*	The indexing component used for dictionary look-up.

Residual component	The remaining part of a character after its radical is removed (Wang et al., 2025).

Stroke pattern 部件 *bu4 jian4*	A stroke-based unit serving as a functional building block in character composition (National Language Commission, 2009).

Stroke 笔画 *bi3 hua4*	The smallest writing unit in regular script (National Language Commission, 2009).

Semantic component 声旁 *sheng1 pang2*	The component in a character that is indicative of its meaning.

Phonetic component 形旁 *xing2 pang2*	The component in a character that is indicative of its pronunciation.


Previous studies on orthographic neighbourhood size in Chinese script have often sidestepped these complexities by treating characters as simple combinations of two components, typically at the radical level, such as radicals and residual components, or semantic and phonetic components. These studies often adopt the one-component substitution method (analogous to Coltheart’s N in the alphabetic writing systems) to define orthographic neighbours. For radical-substituting neighbours, as the non-substituted component can vary widely in visual complexity, neighbours tend to differ greatly in their similarity to the target character. For instance, 凝 *ning2* ‘solidify’ and 冰 *bing1* ‘ice’ share the radical 冫 but they are not orthographically similar overall, due to the varying visual complexity of the residual components. This issue can be partially resolved by defining neighbours using residual components: as they usually occupy more salient parts of characters, the corresponding neighbours tend to be more consistent in their similarity level to the target (e.g., 咤 *zha4* ‘roar’ and 挓 *zha1* ‘expand’ sharing the residual component 宅). However, these methods at the radical level share at least two limitations. They miscateogorise some similar characters as unrelated, such as those that differ below the radical level (e.g., 托 *tuo1* ‘trust’ & 挓) or those that form part-whole relationships (e.g., 刘 *liu2* ‘a common surname’ & 浏 *liu2* ‘clear stream’). More importantly, as neighbours are defined by component inclusion alone, orthographic similarity is reduced to a binary judgment (similar/dissimilar), rather than a graded, continuous measure closer to human perception (Wang and Keuleers, 2024). Ignoring the hierarchy of characters thus results in an imprecise judgment of orthographic similarity and a theoretically compromised account of the effects of orthographic neighbourhood measures. This gap calls for a more appropriate conceptualisation of orthographic neighbourhood measures, which motivates the current paper. We will return to the empirical findings of the neighbourhood size effects in Chinese later; for now, we trace the neighbourhood size research back to its origins in alphabetic scripts.

### Orthographic neighbourhood size effects in alphabetic scripts

Havens and Foote (1963) defined a word as having competitors if there are other words of the same length that differ in only one letter. In a recognition task under tachistoscopic conditions, they found that the presence of competitors inhibited word recognition after word frequency was controlled for. Landauer and Streeter (1973, Study 1) later investigated the relation between the *number* of competitors a word has and its frequency. Rather than using the term competitors, they referred to these words as neighbours and found that more frequent words tend to have more neighbours than less frequent words.

The number of neighbours is now commonly referred to as Coltheart’s **N**. In a lexical decision task, Coltheart and colleagues (1977) divided stimuli into a group with high-N and another with low-N. They found that N had inhibited nonword processing but had no effect on word processing. Andrews (1989) examined the interaction between word frequency and neighbourhood size in a series of lexical tasks using four-letter stimuli. They found a facilitatory effect in lexical decision and speeded naming, but only for low-frequency words. Subsequent studies (Andrews, 1997; Hendrix and Sun, 2021; Holcomb et al., 2002; Pollatsek et al., 1999; Sears et al., 1995; Ziegler and Perry, 1998) confirmed this, although Snodgrass and Mintzer (1993) found inconsistent effects across tasks.

The neighbourhood size effect has also been investigated for other alphabetic languages. In French, Peereman and Content (1995; Study 1) found that N facilitated both speeded and delayed naming, but only for low-frequency words and nonwords. Grainger et al. (1989; Study 1) found the evidence for the neighbourhood frequency effect: words with higher-frequency neighbours were responded to more slowly in lexical decision and progressive demasking paradigms (Grainger and Segui, 1990). Similar studies have also been done for Dutch (e.g., Frauenfelder et al., 1993), Spanish (e.g., Carreiras et al., 1997), German (e.g., Fiebach et al., 2007), Portuguese (e.g., Justi and Jaeger, 2017), and so on. For a more detailed review of N in alphabetic scripts, see for example Andrews, 1997 and Perea, 2015.

Coltheart’s N overlooked word similarity via letter deletions or additions. Yarkoni et al. (2008) then introduced*OLD20*, which measures the average Levenshtein distance to a word’s 20 nearest neighbours (Levenshtein et al., 1966). While these two measures were highly correlated (*r* = –.925) when measuring short, monosyllabic words, OLD20 was advantageous for longer words which typically have neighbours via deletions. In addition, recently, the one-letter distance approach has been used to build orthographic networks (e.g., Siew, 2018), which extended the orthographic analysis beyond local neighbourhoods to the scale of the whole lexicon. These networks are often constructed by representing each word as a node and connecting words that differ by a letter. In such networks, degree, a commonly reported node measure, counts immediate connections and therefore provides complementary evidence to N. Instead of representing words as strings of letters, Tulkens et al. (2018) represented them as vectors and introduced RD20—the average cosine distance to the 20 nearest vectors. They found that RD20, using one-hot encoded words (where each word is represented as a unique high-dimensional binary vector of a fixed length), slightly outperformed OLD20 in explaining variance in lexical decision response latencies across Dutch, English, and French.

Nonwords are usually created with the clear purpose of resembling real words. Keuleers and Brysbaert (2011) noted that such similarity might introduce bias into experiments, as was demonstrated by a nearest-neighbour model that computed the likelihood of recognising a stimulus based solely on its similarity to what was presented before it.

### Orthographic neighbourhood size effects in Chinese script

Studies on two-character words have commonly used the one-character substitution method to determine orthographic neighbours, although they have varied with respect to character position. Huang et al. (2006) defined NS as the number of two-character words that share either the first or the second character with a target word. They found that NS facilitated recognition of high-frequency words but inhibited recognition of low-frequency words in a lexical decision task. In contrast, NS counted on words sharing the first characters has been consistently shown to facilitate lexical decision (Tsai et al., 2006; Xiong et al., 2024), naming (Li et al., 2015), and sentence reading (Tsai et al., 2006). Similarly, NS defined as sharing the second characters also facilitated lexical decision, particularly when neighbourhoods had high semantic consistency (Hsieh et al., 2024; referred to as ‘family size’).

Orthographic neighbours between single-character words are mainly assessed via approaches that rely on sub-character structural units, along with pixel-based approaches that measure visual similarity directly from character images (e.g., Sun et al., 2018). At the finest granularity, strokes are used, and two characters are considered orthographic neighbours if they differ by one or more strokes (e.g., Yu et al., 2023). Using this definition, Shen and Foster (1999) found that characters were named faster if they were primed by neighbours. However, in priming lexical decision tasks, both the presence of neighbours (Wang et al., 2014; Yu et al., 2023) and their average character frequency (Liu et al., 2023) inhibited recognition time. Using ERP data, characters with large neighbourhoods were found to have stronger N400 effects, which are typically associated with the processing of meaning (Dong et al., 2015; Wang and Zhang, 2011). Yet, there is currently no literature addressing the impact of stroke-based neighbourhood size on response latencies in psycholinguistic tasks.

At the radical–residual level, neighbourhood size can be defined in various ways targeting radicals or residual components. Radical *frequency* (or *combinability*) is one neighbourhood size measure. It refers to the number of characters that share a radical, in other words, differ by a residual component). Parallelly, residual component *frequency* (or *combinability*) refers to the number of characters that share a residual component, or, differ by a radical. The broader term subcomponent frequency refers to the size of neighbourhoods defined by either.

Using characters with horizontal structures whose radical and residual component are arranged side-by-side, e.g., 别 (⿰另刂), Taft and Zhu (1997, Study 1) found that the residual component frequency facilitated character recognition in lexical decision, whereas the effect of radical frequency was not significant. In contrast, Wang (2002) reported an inhibitory effect of radical frequency in a component detection task, where frequency by token was used. More recently, Wang et al. (2025) demonstrated a more complex picture, revealing that the effects of radical frequency and residual component frequency depended on character frequency. For frequent characters, both high radical frequency and high residual component frequency slowed down recognition, suggesting competition from orthographic neighbours. For less frequent characters, however, high residual component frequency sped up recognition as radical frequency increased, suggesting that neighbours provide supports. These interactions were observed for both position-general and position-specific subcomponent frequency measures. Collecting EEG data in a lexical decision task, Wu et al. (2012) found that characters with low position-general subcomponent frequency elicited a greater P200 effect, while characters with low position-specific subcomponent frequency elicited stronger P150, P200, and N400 effects. These findings suggest that sublexical processing was involved in both early lexical processing and later semantic processing, while the effect of position-specific subcomponent frequency lasted longer.

The role of radicals and residual components extends beyond their function as orthographic constituents. Approximately 80% of characters are known as phonograms (Yu and Reichle, 2017). A phonogram consists of a semantic component indicating its semantic category (usually a semantic radical) and a phonetic component indicating its pronunciation (usually a phonetic residual component). Feldman and Siok (1997) in a lexical decision task found that semantic radical NS facilitated character recognition, but in a later follow-up study, the authors (1999) found that this facilitation could be task-dependent and time-sensitive. For example, in a primed lexical decision task, the effect disappeared with long lags between characters and their orthographic neighbours sharing a semantic component (e.g., 冷 ‘cold’ and 冰 ‘ice’ both contain the semantic component 冫, which is related to ‘water’). In a naming task with ERP data collected, Hsu et al. (2021) found that, at the early stage of visual element discrimination (N170 time window), the effect of semantic radical NS was significant in reading low-consistency words. The authors explained that the activation of semantic radicals seemed to depend on phonetic components.^2^

The phonetic component NS is defined as the number of phonograms that share a phonetic component (e.g., 澋 and 憬 share the phonetic component 景 and both characters are pronounced as *jing3*). A phonetic component determines two phonological features of its character: *regularity* (whether the pronunciation of the phonetic component agrees with the character’s pronunciation) and *consistency* (to what extent the character’s pronunciation agrees with other characters that share its phonetic component). Li et al. (2020) conducted naming and lexical decision to investigate NS. To account for phonological effects, they selected phonograms that were either consistent-regular or inconsistent-irregular. Their results showed that a larger phonetic component NS inhibited the recognition of both types of phonograms, even after controlling for character frequency and number of strokes. The authors offered two explanations for this: first, the pronunciation derived from the phonetic component was often inaccurate, and second, the presence of more orthographically similar candidates with unreliable phonological values led to confusion at the orthographic level. These findings were in line with Li et al. (2011). For detailed reviews on phonetic component NS, see Hsieh et al. (2021).

### Linearising character composition for neighbourhood measures

Recently, Wang and Keuleers (2024) proposed using Ideographic Description Sequences (IDSs) to compute character similarity. As a standardised system, IDSs encode the compositional structure of CJKV^3^ characters. In the original IDS database (Morioka and Wittern, 2002; kawabata, 2018), an IDS describes a character’s composition specifying its components and the spatial relationships between these components using 12 layout descriptors (⿰⿱⿲⿳⿴⿵⿶⿷⿸⿹⿺⿻).^4^ Each layout descriptor specifies the number of components, mostly two, except for ⿲ and ⿳ which take three, as well as their spatial arrangement. For example, 捌 is described as ⿰扌别, where ⿰ indicates a left-right arrangement of its two components, 扌 and 别. A challenge in using the original IDSs for similarity computation is that characters are decomposed to inconsistent levels of granularity. For example, 别 is described as ⿰另刂, meaning it is decomposed more deeply than 捌 (⿰扌别), making direct similarity comparison between the two challenging.

**Figure 2:**
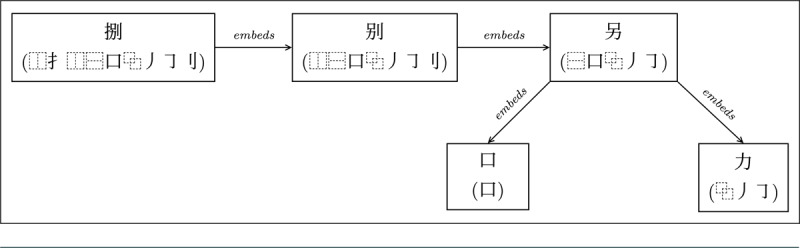
Illustration of nested character structures with their fully decomposed IDSs provided. We show five characters here, three of which embed individual characters. 捌 *ba1* contains 别 *bie2* ‘farewell’, which contains 另 *ling4* ‘other’, which in turn embeds 口 *kou3* ‘mouth’ and 力 *li4* ‘power’.

Wang and Keuleers (2024) addressed this by recursively replacing all components that could be further decomposed with their corresponding IDS representations in the database. As a result, each character obtained a ‘flattened’ representation, to which string-based methods can be applied (see Methods in Study 2). These representations consist of layout descriptors and stroke patterns (Figure 2). The number of stroke patterns in the dataset is 510, including 213 stroke patterns not yet assigned a code point, such as ‘&CDP-8CE6;’, and this size is comparable to the 514 stroke patterns identified in a Chinese national standard (National Language Commission, 2009). The final IDS representations of 20,830 characters used in the paper are provided in Data Availability.

Compared to the radical-based neighbourhood size measures, using IDSs to construct neighbourhood measures directly address their limitations. First, by decomposing characters to the stroke-pattern level, sub-radical similarity can be captured. Second, by applying string-based operations, i.e., substitutions, additions, and deletions, the part–whole relationships can also be captured. Third, by computing distance metrics over the linearised IDS representations, orthographic similarity between characters becomes a graded, continuous measure, more closely approximating human perception. It should be recognised that the stroke-based measures could even represent similarity at an even more nuanced level. Yet, no existing system encodes strokes and their spatial relationships. In addition, given that few characters have neighbours by a single stroke difference, such measures, even if existing, would be difficult to operationalise at the scale of a full lexicon. The IDS-based measures therefore constitute an optimal solution.

However, despite proposing the methods, Wang and Keuleers (2024) have not run statistical models to study how these measures influence character recognition. In what follows, we then present a series of studies in which we predict lexical decision response times (RTs) based on various neighbourhood size measures. First, we attempt to replicate previous findings measuring neighbourhood sizes using semantic and phonetic components. The effects of radical frequency and residual component frequency have been addressed in a separate study (see Wang et al., 2025). Second, we propose a set of distance-based neighbourhood size measures using characters encoded as IDSs.

## Study 1: Replication of semantic component NS and phonetic component NS

In Study 1, in line with previous practices, we used phonograms because their semantic-phonological composition allowed for clear quantification of neighbourhood size. We modelled lexical decision response times using neighbourhood size measures based on semantic and phonetic components, while controlling for established lexical and phonological variables. We used a linear mixed-effects model (LMM) for the analysis.

### Method

#### Design

Table 2 shows the independent variables and their definitions. To obtain neighbourhood size measures and the consistency variable, we used phonograms documented in the Chinese Lexical Database (Sun et al., 2018; 3,146 characters labelled as ‘PicPho’ for ‘C1Type’). Non-phonograms and missing values were removed, resulting in a total of 3,137 phonograms. The 1,020 phonetic components and 186 semantic components extracted from these phonograms were then used to compute consistency and neighbourhood size measures.

**Table 2:** Definition of the independent variables used in Study 1. Consistency and regularity were included to control for potential phonological influence. Nstrokes and character frequency were included as they are strong predictors of word recognition performance.


VARIABLE	DEFINITION

N-sc	number of phonograms sharing a semantic component with a target

N-pc	number of phonograms sharing a phonetic component with a target

consistency	number of characters sharing the same pronunciation (regardless of tones) divided by the total number of characters sharing the same phonetic components

regularity	a binary measure encoding whether the phonetic component’s pronunciation agrees with the pronunciation of the character (regardless of tones)

NStrokes	number of strokes retrieved from online Xinhua Dictionary^5^

character frequency	character frequency calculated as Zipf value according to Van Heuven et al. (2014)


In addition, we used the Zipf value to represent character frequency to address the issue of unobserved characters (Van Heuven et al., 2014). Data for calculating the Zipf value were retrieved from the SUBTLEX-CH (Cai and Brysbaert, 2010) and the equation was:


$$Z_{\text{ipf value}}=\log_{10}\left(\frac{\text{rawcount}+1}{48.6+0.015}\right)+3$$

where the raw count was the occurrence of a character documented in SUBTLEX-CH (Cai and Brysbaert, 2010), which was 0 for an unobserved character. The value 48.6 represented the total number of characters in the corpus in millions. The value 0.015 represented the estimated number of unobserved character types in millions, based on character sets in the corpus (*N* = 5,936) and in the online Xinhua Dictionary (*N* = 20,830).

#### Material

Response times for characters were retrieved from the Simplified Chinese Lexicon Project (SCLP; Wang et al., 2025), which collected trial-level lexical decision response measures for 8,105 characters and 4,864 pseudocharacters from 29 native speakers. In line with the study, we removed incorrect responses, responses with reaction times outside the scope of three times the interquartile range, responses faster than 200 ms or slower than 1,500 ms, and items with a mean accuracy equal to or below 67%. Table 3 shows a complete overview of these steps. Note that the large number of characters with low accuracy reflects the fact that many characters are rarely used and thus known by few people. After the data cleaning procedure, data for 4,528 characters with 115,518 trials were retained (hereafter **clean characters**).

**Table 3:** The criterion for removal at each step of data cleaning. Incorrect responses, IQR outliers, and RT outliers were removed at the trial level; Mean accuracy was assessed at the character level. The number of trials and characters removed is provided.


DATA CLEANING STEPS	NUMBER OF TRIALS			NUMBER OF CHARACTERS		
	
	BEFORE	REMAINING	REMOVED	BEFORE	REMAINING	REMOVED

Raw data	235,045			8,105		

Incorrect responses	235,045	144,079	90,966	8,105	7,745	360

IQR outliers (±3 IQR)	144,079	137,766	6,313	7,745	7,680	65

RT outliers (< 200 ms or > 1,500 ms)	137,766	134,204	3,562	7,680	7,650	30

Low accuracy (<= 67%)	134,204	115,518	18,686	7,650	4,528	3,122

*Clean character set*		**115,518**			**4,528**	


Of these clean characters, data for all the independent variables were available for 2,767 characters (70,534 trials), which were used for the subsequent statistical analysis. See Table 4 for descriptive statistics of the variables.

**Table 4:** Descriptive statistics of the continuous variables used in Study 1. For the variable regularity, there are 1,410 regular characters and 1,350 irregular characters.


VARIABLE	FIVE-NUMBER SUMMARY				

	MIN.	Q1	MEDIAN	Q3	MAX.

RT	341.010	583.355	657.088	771.030	1499.745

N-sc	1	25	63	140	224

N-pc	1	3	5	8	17

consistency	.059	.333	.556	1.000	1.000

NStrokes	3	8	10	13	25

character frequency	1.614	3.374	4.111	4.783	7.559


#### Data Transformation and Model Selection

Response times were inverse-transformed using -1000/x to reduce data skew (Baayen and Milin, 2010). For model selection, we first built a raw model with character frequency, NStrokes, N-sc, N-pc, regularity, and consistency as fixed effects factors and with random intercepts for item and subject. Next, we removed residual outliers beyond ± 2.5 standard deviations. Finally, we refitted the resulting model.

#### Data Analysis and Visualisation

For the studies presented in this paper, we used the *levenshtein* package (Bachmann, 2025) to compute Levenshtein distance in *Python* (Python Software Foundation, 2024). The statistical analyses were conducted using *R* (Ihaka and Gentleman, 1996). We used the *lme4* package (Bates et al., 2015) and the *lmerTest* package (Kuznetsova et al., 2017) to run linear mixed-effects models. *P*-values for fixed effects factors were estimated using Satterthwaite’s method. Other packages included *r2glmm* (Jaeger, 2017) for obtaining marginal *R*^2^ (method = ‘nsj’), *car* (Fox and Weisberg, 2019) for obtaining variance inflation factors (VIFs), *psych* (William Revelle, 2025) for performing principal component analysis (PCA), and *ggplot2* (Wickham, 2016) for plotting.

### Results

Table 5 shows the correlations between response times and predictors. While response times exhibited a moderate correlation with frequency and NStrokes, their correlation with the other measures was limited. A moderate correlation between N-pc and consistency was found (*r_s_* = –.579): consistency decreases as neighbourhood size increases. Consistency also had a moderate correlation with regularity (*r_s_* = .516): consistency increases as regularity increases. Both consistency and regularity relate to the pronunciation of phonetic components. Regularity measures the agreement between a character and its phonetic component, while consistency measures the between-character agreement on shared phonetic components.

**Table 5:** Spearman’s rank correlations between response times and independent variables for characters (*N* = 2767) used in the replication of neighbourhood size effects based on semantic components and phonetic components.


	RT	CHARACTER FREQUENCY	NSTROKES	N-SC	N-PC	REGULARITY	CONSISTENCY

RT	1.000	–.289	.164	.012	–.038	.023	.057

character frequency		1.000	–.215	–.038	.044	–.069	–.107

NStrokes			1.000	–.129	–.159	.031	.159

N-sc				1.000	–.127	.086	.151

N-pc					1.000	–.149	–.579

regularity						1.000	.516

consistency							1.000


Table 6 shows the facilitatory effects of character frequency and the inhibitory effect of NStrokes. None of the other variables seemed to strongly affect reaction times. To assess collinearity among the phonological variables (Dormann et al., 2013), we computed the variance inflation factors (VIFs; O’brien, 2007) for the predictors in the LMM. Common cutoffs for serious collinearity include 10 and 5 (see, for example, Kutner et al., 2004; O’brien, 2007), while Graham (2003) shows that a VIF as low as 2 already suggests considerable collinearity. Although the VIF of consistency is close to the conservative cutoff of 2, we still accepted the possible mild variance inflation in favor of its theoretical importance and thus retained the variable in the model. To address concerns of collinearity, we applied PCA with varimax rotation, following the approach of Hendrix and Sun (2021). The results are provided in the Appendix.

**Table 6:** Fixed effects results from the LMM in Study 1 (fitted to 2,767 characters and 69,370 trials). The variance inflation factors, marginal *R*^2^, and 95% confidence intervals with lower and upper limits are provided.


VARIABLE	ESTIMATE	STD. ERROR	*DF*	*T*	*P*	VIF	MARGINAL *R*^2^

(Intercept)	–1.278	0.027	54.261	–46.552	<.001***	—	—

character frequency	–0.087	0.002	2752.663	–42.455	<.001***	1.059	.076 [.072, .078]

NStrokes	0.012	0.001	2738.669	17.903	<.001***	1.114	.015 [.013, .016]

consistency	0.011	0.009	2717.062	1.253	.210	1.963	.000 [.000, .000]

regularity	–0.002	0.005	2722.966	–0.408	.683	1.415	.000 [.000, .000]

N-sc	0.000	0.000	2715.690	1.642	.101	1.050	.000 [.000, .000]

N-pc	–0.000	0.001	2711.059	–0.259	.795	1.458	.000 [.000, .000]


#### Discussion

We found that recognition latency decreased with character frequency, consistent with the literature on the effects of word frequency in general (e.g., Brysbaert et al., 2018) and character frequency in particular (e.g., Wang and Keuleers, 2024). In line with earlier findings (e.g., Sze et al., 2014; Wang et al., 2025), we also found an inhibitory effect for number of strokes. On the other hand, consistency and regularity showed no effect, in line with Yum et al. (2014) but contrary to the facilitatory effects reported by, e.g., Li et al. (2011). For neighbourhood size measures, we found no effect of N-sc. Radicals often convey semantic information so we compare the effect of N-sc with the effect of radical frequency. This null effect of N-sc is consistent with, e.g., Taft and Zhu (1997), but is inconsistent with some others (e.g., Wang et al., 2025).

Prior studies of neighbourhood size effects mainly used phonetic components and found a mixture of results (e.g., Hsieh et al., 2024; Taft and Zhu, 1997). Taft and Zhu (1997) showed that characters with larger neighbourhoods based on residual components were recognised faster. Since these residual components were located on the right side of the characters, they accordingly suggest an important role of positioning in character recognition. However, Feldman and Siok (1997) manipulated the positions and functions of subcomponents and found similar facilitatory effects, but argued that these stemmed mainly from the *semantic* and *phonological* function of the radical-level components rather than their positioning. In contrast, Li et al. (2020) reported an inhibitory effect of neighbourhood size, mainly on phonetic components, and their interpretation pointed to possibly inaccurate pronunciation cues from phonetic components or confusion at the orthographic level due to high component overlap. Adding to the inconsistency, null effects of neighbourhood size based on phonetic components were also reported (e.g., Wang et al., 2025). In the present study, we found no effect of N-pc.

This result pattern then suggests that the presence and direction of these neighbourhood effects are less robust than one might hope for. In contrast, it could be dependent on ‘local’ factors, such as stimulus selection, specific sources used to calculate lexical measures, and model architecture. An alternative explanation relates to the nature of the task used. For example, Li et al. (2020) found a robust N-pc effect in a naming task, significant by subjects and by items, but the inhibitory effect in their lexical decision task was only significant by subjects. The present study used lexical decision, and N-pc and N-sc may more sensitive to tasks that put higher demand on semantic or phonological processing. For N-sc, an example could be semantic judgment, where participants identify whether two characters share the same semantic category; for N-pc, an example task could be naming, where participants read out presented stimuli.

An important shortcoming is that, until now, the measurement of orthographic neighbourhood size has relied on a simple dichotomy (i.e., semantic vs. phonetic, or, more generally, radical vs. residual). As described earlier, semantic components are often a simple and small part of a whole character, while phonetic components tend to be larger and more complex. This makes it difficult to conceptualise characters sharing semantic components as clear orthographic neighbours. On the other hand, although phonetic components tend to play a much larger role in characters’ similarity, their often inconsistent mapping to pronunciation makes it hard to separate the effects of phonology from orthography. As measures, N-pc and N-sc do not capture similarity between: (1) characters that differ by finer-grained subcomponents (e.g., 摒 and 拼), (2) characters with identical components arranged in reverse positions (e.g., 杏 and 呆), and (3) characters in a part–whole relationship (e.g., 刘 and 浏).^6^

These limits call for an alternative method of character representation that can define orthographic similarity more precisely. The introduction of Ideographic Description Sequences (Unicode Consortium, 2023) has provided a possible solution (Wang and Keuleers, 2024), as they encode fine-grained stroke patterns and their spatial relations. This systematic, linear representation also allows for the use of established string-based similarity measures that are typically used in identifying similar words in alphabetic languages (e.g., Yarkoni et al., 2008).

## Study 2: Implementation of IDS-based neighbourhood measures for Chinese characters

In Study 2, we used IDS representations for characters and assessed the effects of neighbourhood measures operationalised as N-ch, WLD-10, and WND-10 on lexical decision response times.

### Method

#### Design

In the representation of Chinese characters, an IDS starts with a layout descriptor followed by arguments, which can be either stroke patterns or another layout descriptor. For example, the IDS of 英 *ying1* ‘blossom’ is represented as ⿱艹央 and that of 嚻 *xiao1* ‘a mythic beast’ is ⿲⿱口口頁⿱口口 (examples from Wang and Keuleers, 2024).

Analogous to Coltheart’s N (Coltheart et al., 1977), we introduce N-Chinese (N-ch). **N-ch** is defined as the number of characters that differ from a target character by the substitution of one component (either a layout descriptor or a stroke pattern). For example, character 器 has a N-ch of 4, pointing to its four neighbours by one substitution (噐, 嚚, 嚣, and 囂).

**Figure 3:**
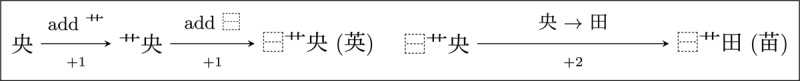
Illustration of edit distance calculation under the weighted scheme. Both pairs (央 & 英 and英 & 苗) have a distance of 2.

Parallel to Yarkoni’s OLD20 (Yarkoni et al., 2008), we introduce WLD-k. **WLD-k** is defined as the mean of the weighted Levenshtein distance between a character and its *k* nearest neighbours. In the current implementation, we assign a cost of 2 to a *substitution* and a cost of 1 to an *addition* or *deletion*. This corrects an asymmetry in the calculation of raw distance that arises from the use of IDS representations. In an IDS representation, adding or deleting a stroke pattern affects both the component set and the layout of a character. For example, to add a stroke pattern 艹 to 央 (IDS: 央), we must first specify their spatial relationship with a layout descriptor. With ⿱, this produces 英 (IDS: ⿱艹央) and the distance between the two characters is 2. However, replacing the bottom component of 英 to form 苗 counts as a single substitution, leading to a distance of only 1. This unweighted scheme thus treats characters with a subpart–whole relationship (i.e., 英 vs. 央) *less similar* than characters differing by a substitution (i.e, 英 vs. 苗), although both character pairs differ by one stroke pattern perceptually. Weighting substitution at a cost of 2 while keeping addition and deletion at a cost of 1 resolves this issue, ensuring that swapping a component and adding/deleting a component result in equal distance. In the above example, both pairs end up having the same distance of 2 using the weighted scheme (see Figure 3 for an illustration of the calculation steps).

**Figure 4:**
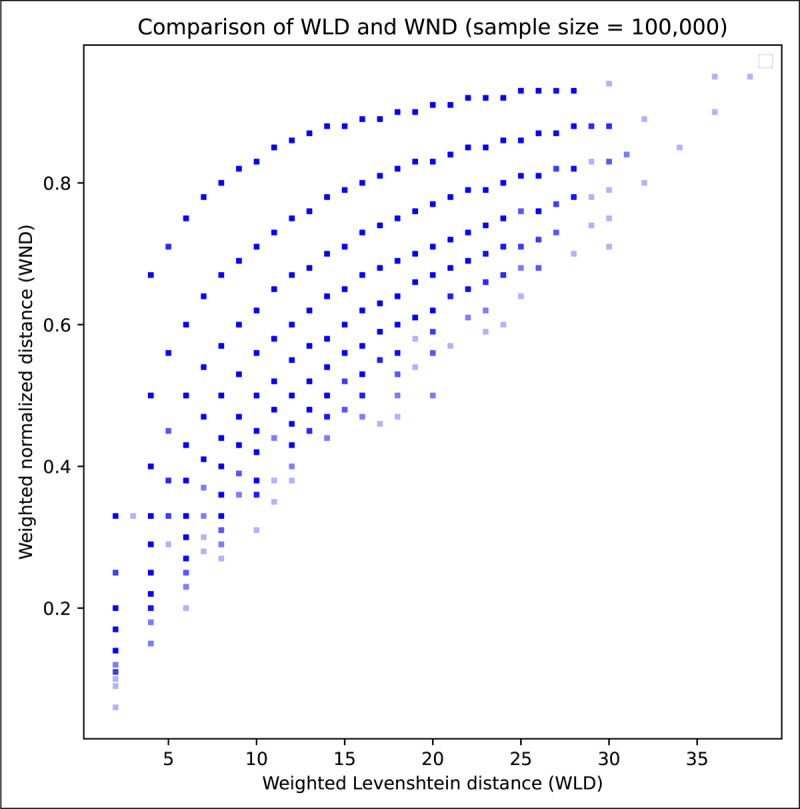
Comparison of weighted Levenshtein distance (WLD) and weighted normalised distance (WND). For each character, its distance from the full dataset (20,830 characters) was identified separately under each measure. A total of 100,000 characters were randomly sampled, with coordinates of each dot showing WLD and its corresponding WND.

Levenshtein distance is an absolute measure of the number of operations required to transform one sequence into another. It is preferable to use a normalised version of edit distance, because absolute edit distance is not independent of sequence length (Wang and Keuleers, 2024). As Figure 4 shows, different pairs of characters with the same absolute distance can occur at widely differing normalised distances: the same absolute distance becomes a smaller normalised distance as the sequence length of the pairs increases. Table 7 illustrates this further, by comparing absolute and normalised distances for a few examples in English and Chinese.

**Table 7:** Comparison of absolute and normalised edit distance measures. The steps of WLD calculation for the English word examples are illustrated in Figure 5. Chinese characters: 鬰 *yu4* ‘lush and growing abundantly’, 礬 *fan2* ‘alum’, and 匕 *bi3* ‘spoon’. The IDS of 鬰 is ⿳⿲木⿱㐅⿻丿乀 木冖⿰⿱⿶凵⿻ 㐅⿳丶⿰丶 丶丶⿺乚丿彡, of 礬 is ⿱⿱⿲ 木⿱㐅⿻丿乀木⿻一人⿸⿱一 丿口, and of 匕 is ⿺乚丿.


	WEIGHTED LEVENSHTEIN DISTANCE	WEIGHTED NORMALISED DISTANCE

*D*(hair, pen)	**7**	1.000

*D*(hair,hailstone)	7	**.540**

*D*(鬰, 匕)	**23**	.793

*D*(鬰, 礬)	24	**.545**


**Figure 5:**
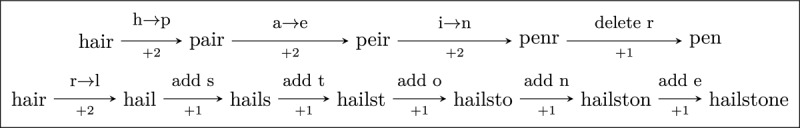
WLD calculations for two English word pairs. Each has a distance of 7.

Following Wang and Keuleers (2024), normalised edit distance is calculated by dividing the absolute Levenshtein distance between two strings by the maximum possible distance between them. Given a weighted Levenshtein distance as used in WLD-k, the corresponding weighted maximum distance is:


$$\text{max distance}=\min\left(N_{i},N_{j}\right)\times 2+\left|N_{i}–N_{j}\right|$$

where $N_{i}$ and $N_{j}$ are the lengths of IDSs of characters $C_{i}$ and $C_{j}$ . The resulting weighted normalised distance has a range from 0 (characters are identical) to 1 (characters have no shared components in their IDSs).

#### Material, Data Transformation, and Model Selection

To obtain N-ch, WLD-k, and WND-k for characters, we retrieved the IDS representations for the characters in the Xinhua Dictionary (*N* = 20,830), from an online repository (Morioka and Wittern, 2002; kawabata, 2018). We then obtained representations for each character (Wang and Keuleers, 2024), based on which all three measures were computed. For *k* in WLD-k, we tested all values from 5 to 50 with identical model architecture and selection procedure as described below. The WLD-k coefficient was significant across a wide range (5 to 45), and the highest t-values were observed with k between 7 and 12 (4.47 to 4.72). We chose a whole number in this optimal range (*k* = 10), in line with the value of 20 in OLD-20. To mirror this, we used the same number for WND-k. Table 11 shows two example characters with WLD-10 and WND-10.

Out of all the clean characters (*N* = 4,528; see Study 1), we removed those with only one component in their IDS (N = 144), such as the character 一 *yi1* ‘one’, since by definition, all other one-component characters are neighbours but completely different. This left 4,384 characters and a total of 111,518 trials for analysis. See Table 8 for the descriptive statistics.

**Table 8:** Descriptive statistics for variables used in Study 2.


VARIABLE	FIVE-NUMBER SUMMARY				

	MIN.	Q1	MEDIAN	Q3	MAX.

RT	298.450	574.375	649.328	764.340	1499.745

Character frequency	1.313	3.243	4.141	4.898	7.559

NStrokes	2	8	10	12	25

N-ch	0	1	5	13	117

WLD-10	1.800	2.000	2.000	3.200	11.300

WND-10	.060	.151	.200	.265	.619


We ran three LMMs to predict lexical decision response times: Study 2a for N-ch, Study 2b for WLD-10, and Study 2c for WND-10. The architecture of these models was identical except for the neighbourhood measure. Specifically, each model included character frequency, NStrokes, and one neighbourhood measure as fixed effects factors, with random intercepts for item and subject. The data transformation and model selection remained unchanged from Study 1. The dependent variable (response times) was transformed using –1000/x; after fitting the initial model, we removed residual outliers and the resulting data were used to refit the final models. The number of characters and trials per model is provided in the Results section.

### Results

Table 9 shows the correlations between the predictors and response times. Both N-ch and WLD-10 were derived from the absolute Levenshtein distance and they were inversely related: larger N-ch reflects a **higher** degree of orthographic similarity to others through the larger size of immediate neighbourhood in the orthographic lexicon, while larger WLD-10 reflects **lower** orthographic similarity through longer distance to its ten nearest characters. Reflecting this, they were strongly negatively correlated and were therefore used for separate models. In comparison, although the normalised neighbourhood density measure WND was computed based on WLD, these two measures had a very low correlation, suggesting that they either capture different neighbourhoods altogether, or characterise similar neighbourhoods with different levels of nuance. Similarly, WND-10 had a negligible correlation with N-ch.

**Table 9:** Correlations between variables used in Study 2 (Spearman’s r).


	RT	CHARACTER FREQUENCY	NSTROKES	N-ch	WLD-10	WND-10

RT	1.000	–.351	.236	–.025	.120	–.029

Character frequency		1.000	–.286	.040	–.149	.055

NStrokes			1.000	–.253	.363	–.199

N-ch				1.000	–.770	.007

WLD-10					1.000	.236

WND-10						1.000


Like Study 1, the correlations between neighbourhood size measures (N-ch, WLD-10, and WND-10) and response times were weak. Of note, however, is that the correlation between NStrokes and WND-10 was smaller and had a different sign than the correlation between NStrokes and WLD-10. This suggests that WLD-10 partly reflects the number of strokes, with higher values being more likely for characters with more strokes. WND-10, on the other hand, should be insensitive to number of strokes. The smaller correlation between WND-10 and RT does not mean that WND-10 is a worse measure of neighbourhood density. Rather, it is a purer version of neighbourhood density, which incorporates aspects of neighbourhood density as well as character length.

Table 10 shows that character frequency had a facilitatory effect and NStrokes had an inhibitory effect and the pattern remain unchanged across models. All neighbourhood measures had reached significance.^7^ To check for collinearity, as in Study 1, we calculated the VIFs and the results imply that collinearity was not a large concern for all models. However, the results for marginal *R*^2^ show that these neighbourhood measures explained only a minimal share of the total variance in the response times (at most 0.01%), despite being statistically significant.

**Table 10:** Results for fixed effects factors across the LMMs in Study 2. For model architectures, the three models only differed in the neighbourhood measure used (initial N = 4,384 characters, 111,518 observations). After removing residual outliers, the number of characters remained unchanged for all models and the final observation counts were similar across models (2a: 109,632; 2b: 109,631; 2c: 109,630).


ID	VARIABLE	ESTIMATE	STD. ERROR	*DF*	*T*	*P*	VIF	MARGINAL R^2^

Study 2a	(Intercept)	–1.332	0.025	37.841	–53.485	<.001***	/	/

	Character frequency	–0.089	0.001	4402.104	–60.589	<.001***	1.083	.104 [.100, .107]

	NStrokes	0.017	0.001	4364.659	31.137	<.001***	1.147	.030 [.028, .032]

	N-ch	0.001	0.000	4332.447	6.531	<.001***	1.062	.001 [.001, .002])

Study 2b	(Intercept)	–1.330	0.025	38.286	–53.239	<.001***	/	/

	Character frequency	–0.088	0.001	4401.886	–60.350	<.001***	1.085	.103 [.100, .107]

	NStrokes	0.015	0.001	4359.706	26.732	<.001***	1.242	.023 [.021, .024]

	WLD-10	0.008	0.002	4377.645	4.550	<.001***	1.172	.001 [.000, .001]

Study 2c	(Intercept)	–1.338	0.025	41.372	–52.557	<.001***	/	/

	Character frequency	–0.089	0.001	4402.396	–60.599	<.001***	1.082	.104 [.101, .108]

	NStrokes	0.016	0.001	4373.708	30.538	<.001***	1.123	.029 [.027, .031]

	ND10	0.087	0.023	4329.019	3.811	<.001***	1.041	.000 [.000, .001]


A reviewer raised a concern regarding different control variables and character sets used across Studies 1 and 2, which may compromise direct comparisons between the radical-based and the IDS-based neighbourhood measures. To ensure differences in results cannot be attributed to differences in the data sets, we refitted three models of Study 2 (for N-ch, WLD-10, and WND-10, respectively) on the Study 1 dataset, with identical control variables (i.e., regularity, consistency, character frequency, and NStrokes). Despite the presence of additional phonological variables, the effects of IDS-based neighbourhood measures remained statistically significant, showing qualitatively similar results to what was reported above (See Appendix for full model results).

### Discussion

In this study, we used Ideographic Description Sequences to represent characters. Unlike N-pc and N-sc in Study 1, where each character was represented by a combination of two subcomponents, characters using IDS representations encode fine-grained stroke patterns and their spatial relations. As a result, character similarity and orthographic neighbourhood can be more precisely measured.

Both N-ch and WLD-10 were computed based on absolute distance and showed inhibitory effects in the models. As WLD-10 averages distance, its larger values indicate that a character is further from other characters, which means it has greater orthographic distinctiveness. Its inhibitory effect therefore implies a **facilitatory** *density* effect: characters in denser neighbourhoods (lower WLD-10) are recognised faster. This may seem contradictory at first glance, given the inverse relation between the two measures, but they capture different aspects of orthographic similarity. N-ch tells us the size of the **immediate** neighbourhood. In this neighbourhood, neighbours are highly similar to a target. They differ by only one stroke pattern from the target, or they share the same set of stroke patterns but differ in structural arrangement. More neighbours slow down recognition, suggesting possible competition due to high similarity in visual forms. In contrast, WLD-10 tells us how *dense* a character’s region (constrained by a size of 10) is in the whole orthographic lexicon. Our finding suggests that characters more isolated in the hypothetical space are recognised more slowly, while characters embedded in a denser region are faster to process, possibly due to faster global access.

The role of WLD-10 is, in theory, particularly meaningful for complex characters, which tend to have relatively few one-substitution neighbours. Table 11 illustrates this distinction using two complex characters (Nstrokes of around 15) that have the same N-ch of 4 but differ in WLD-10. One character has a higher WLD-10 value of 3.3 (above Q3 = 3.2), indicating higher orthographic distinctiveness beyond its immediate neighbours. The other has a lower value of 2 (equal to the median), indicating that it is located in a denser cluster and is therefore less distinctive even beyond its immediate neighbours.

**Table 11:** Ten nearest characters for two example characters 憬 (left, lower WLD-10) and 器 (right, higher WLD-10). Both have an N-ch of 4, pointing to the first four characters listed in both columns that were formed by one substitution of one stroke pattern from each character. Character 憬 (NStrokes = 15, WND-10 = .103) occupies a denser orthographic neighbourhood beyond its N-ch neighbours, while character 器 (NStrokes = 16, WND-10 = .242) occupies a sparser neighbourhood. Our results show that characters like 憬 with denser neighbourhood are easier to recognise, after controlling for character frequency and number of strokes.


憬 (WLD-10 = 2.0)		器 (WLD-10 = 3.3)		

CHARACTER	DISTANCE	CHARACTER	DISTANCE

澋	2	噐	2

幜	2	嚚	2

暻	2	嚣	2

撔	2	囂	2

晾	2	噪	5

惊	2	嘽	5

景	2	吅	5

颢	2	品	5

影	2	嘔	5


An alternative explanation relates to different types of neighbours included to compute these two measures. Levenshtein distance includes three operations: substitution, addition, and deletion. N-ch only counts the number of neighbours by substitution, ignoring neighbours formed by the other two operation types. By contrast, WLD-10 employs all three and thus reflects an additional whole-subpart relationship between neighbours (Table 11 shows an example with 憬 and 景). The different effects then possibly suggest that orthographic relationships may point to different aspects of orthographic processing, which requires further investigation.

We also introduced a normalised measure of neighbourhood density, WND-10. By using proportional distance to compute neighbourhood density, WND-10 avoids the problem that, for instance, a one-component difference between two-component characters equals a one-component difference between four-component characters. In doing so, it becomes less affected by the character complexity, as can be seen the correlations and VIFs from both models. Despite the conceptual difference, we found a similar result pattern as for WLD-10, with recognition times for characters increasing as a character’s neighbourhood becomes less dense. The substitution of WLD-10 by WND-10 also did not alter the effect of the other variables. These results do not indicate that both measures are interchangeable or equally valid. They do show that, even with a conceptually better measure, neighbourhood density effects remain weak. In addition, while the three measures in the present study capture a character’s level of orthographic distinctiveness in the lexicon, they are still *local* measures without a clear mechanism showing how the *global* organisation of characters shapes their influence. Conceptualising such a clear global organisation of characters based on orthographic relations, though currently outside the scope of this paper, constitutes our future research direction.

## General Discussion

In the present paper, we investigated orthographic neighbourhood effects in Chinese character recognition.

In Study 1, we compared neighbourhood measures based on semantic (N-sc) and phonetic (N-pc) components. These most closely resemble existing methods of defining orthographic neighbours that rely on dichotomous units (e.g., radicals, or phonetic components). We found no effect of either measure on character recognition. As discussed in Study 1, there are a mixture of findings for both measures, and our findings add to the inconsistency in the existing literature. These divergences are likely due to extraneous factors such as stimulus selection, different sources of measures and model architecture. An alternative explanation relates to the nature of tasks used across studies; therefore, more tasks (such as naming) should be tested in order to assess the generalisability of the present findings. More importantly, however, the null result pattern does not eliminate the possibility of orthographic neighbourhood size effects in Chinese character recognition. After all, these measures in Study 1 reflect coarse character compositions, which tend to produce imprecise measurements of orthographic similarity.

In Study 2, we addressed the issue by traversing down the hierarchy of characters and targeted stroke pattern as the sub-character unit to compare character similarity. The Ideographic Description Sequences encoded stroke patterns, as well as their layout descriptors, and thus offered an ideal approach. Using the IDSs, we developed three neighbourhood measures (N-ch, WLD-10, and WND-10). Conceptually, these measures assess orthographic similarity at a more nuanced level. Accordingly, they revealed the significant roles of orthographic neighbours, while the radical-based measures did not show significant effects. The findings remained robust after aligning the stimulus selection and model architecture between Study 1 and Study 2. A cautious interpretation is that, compared to N-sc and N-pc, the IDS-based measures provide a purer assessment of orthographic similarity by avoiding the conflation with semantic and phonological activations. For N-sc, neighbours are defined by shared semantic radicals, which means they also tend to form semantic clusters, i.e., characters close in meaning (e.g., 打 *da2* ‘beat’, 推 *tui1* ‘push’, and 拉 *la1* ‘pull’ all share 扌, meaning ‘hand-related action’). By contrast, the IDS-based operationalisation largely excludes such semantic clustering from the neighbourhood, restraining the influence of semantic activation. The case for phonology is more subtle. Phonetic components are visually more salient than semantic components, so IDS-based neighbours may still incidentally include characters sharing phonetic components, which could provide indirect phonological activation. However, by not explicitly grouping neighbours by phonetic components, the IDS-based measures still reduce the potential confound compared to N-pc.

For the measures in Study 2, both N-ch and WLD-10 were derived from absolute distance metrics and showed inhibitory effects in the models. For N-ch, this is straightforward: more orthographic neighbours slow down character recognition. As these neighbours either differ by only one stroke pattern or differ by a layout arrangement of identical stroke patterns, they were orthographically *highly* similar. Such level of similarity seems to produce competition among characters when identifying the target. For WLD-10, however, the interpretation requires one additional step: as the WLD-10 averages distance to neighbours, a larger value means that neighbours are more distant, i.e., the character occupies a sparser neighbourhood in the whole orthographic space. Its inhibitory effect therefore means that characters in sparser neighbourhoods are recognised more slowly. Or, characters in denser neighbourhoods are recognised faster. This pattern retained with a normalised version that accounted for sequence length (WND-10). Altogether, these findings present a **facilitatory density effect** in contrast to an **inhibitory size effect**, highlighting complicated roles of orthographic neighbours.

An alternative explanation is that the diverging results reflect differences in the distance operations used in computing the size and density measures. N-ch counted neighbours based on substitutions but WLD-10 and WND-10 were computed involving neighbours based on two more operations: additions and deletions. This means that the density measures capture part–whole relationships, which seems to allow a character to benefit from either being a frequent component for more complex characters or containing common characters as its components, or both. Whether this mechanism underlies the density effect observed in the present study requires further empirical evidence. For now, the results imply that if any meaningful effect of neighbourhood measures exists for single character recognition, its nature is probably mixed. Interestingly, this mixed effect is not an isolate phenomenon unique to the Chinese script. Yarkoni et al. (2008) found the same pattern in English word recognition, i.e., the presence of both facilitatory effects of orthographic density (OLD20) and inhibitory effects of orthographic size (N) for lexical decision. The authors reconcile such contrast by suggesting that OLD20 reflects global similarity which benefits a character at the early stage of recognition, while N reflects local similarity which creates competition during final word identification. Our findings suggest that this pattern could be cross-script, and more importantly, this pattern can only be revealed by using a linearised representation of characters (i.e., IDSs) that allows such high resolution of comparison.

At first glance, these effects of neighbourhood measures based on stroke patterns suggest that Chinese readers seem to be sensitive to orthographic information at the level of stroke patterns. This is possibly linked to how the written forms of characters are acquired: as young Chinese readers learn characters initially by rote, they tend to develop sensitivity to fine-grained character configurations. Such sensitivity to orthographic regularity may be acquired surprisingly earlier than one might imagine. Tong and McBride (2014) found that even kindergartners developed awareness of stroke patterns and their positional constraints: they were able to piece up invented characters by allocating stroke pattern pairs in correct positions. This ability improved across grade groups (2nd and 5th grades). One might therefore expect this sensitivity to imply stronger neighbourhood effects. However, even after improving the neighbourhood measures through IDS representations, inclusion of diverse orthographic relations, and normalisation of measures, the effects remained small. For comparison, while these measures (N-ch, WLD-10, and WND-10) explained at maximum 0.1% of variance of lexical decision response times, Yarkoni et al. (2008) reported the equivalent figure of 2% for English (OLD20). Therefore, rather than concluding that stroke patterns may act as perceptual units (e.g., Chen, 1996), we interpret our findings with caution as evidence that by selecting an appropriate level of sub-character components, the IDS-based measures represent a purer assessment of orthographic similarity and reveal how orthographic similarity influences character recognition. These findings also highlight that recognising the hierarchical nature of Chinese characters is crucial for the study of character recognition.

Across scripts, there is a mixture of results for the effects of orthographic neighbourhood size in both direction and magnitude. Studies using English suggest both facilitation (e.g., Andrews, 1989, 1997) and inhibition (Yarkoni et al., 2008), in comparison with the inhibitory effects found in languages such as Portuguese (e.g., Justi and Jaeger, 2017), Spanish (e.g., Carreiras et al., 1997) and French (e.g., Peereman and Content, 1995; Grainger and Segui, 1990). The present study provides little evidence of strong effects of orthographic neighbourhood size for Chinese characters. This difference points to a possibility that the orthographic neighbourhood size effects observed in alphabetic languages are mainly *phonological* neighbourhood size effects. Phonological similarity, instead of lexical similarity at a purely orthographic level, impacts word recognition, and this effect is conditioned on grapheme-phoneme correspondences. When the grapheme-phoneme mappings are consistent and predictable, as is in the case of transparent orthography in French, Spanish, and Portuguese, the orthographic overlap with neighbours inhibits the recognition of a word. When the mappings are less consistent and predictable, as in English, such effects seem to be reversed. Chinese is a morphosyllabic writing system in which each character represents a morpheme, and there are no grapheme-phoneme correspondences. In this case, the orthographic neighbourhood effect in Chinese appears to depend on how neighbourhood size is operationalised. Although orthographic neighbourhood size could be a useful conceptual construct for character recognition, the current results suggest that its effects are small and not robust at best.

There are some limitations to the present study. First, in our implementation of similarity measures, we used weighted edit distance to reflect the fact that the addition/deletion of a stroke pattern updates layout descriptors in the IDS representations. However, for character pairs involving characters with three-element layout descriptors (e.g., ⿲, ⿳), their distances can be artificially inflated. This points to a broader limitation: Ideographic Description Sequences are used to represent the hierarchical structure of characters, which we falsely treated as flat sequences when calculating distances. In future work, we suggest the use of tree edit distance measures, which will probably produce more accurate results. Next, the choice of *k* in WLD-k and WND-k is arbitrary, although the practice is consistent with previous studies. A better solution for studying neighbourhood size effects would be a measure that merges the angle of *size* and *density*: one that combines gradable constraint on defining a neighbour and uses a threshold to maintain comparable similarity levels within neighbourhoods. Third, we remove one-component characters from the analyses. A more proper method would be to remove the neighbour status between any two one-component characters. This would allow us to maintain neighbour status from adding or deleting components, thus retaining more character pairs with whole-part relationships. Fourth, the current analysis is limited to lexical decision latencies. This means that task-specific effects are not addressed, potentially hindering a more comprehensive evaluation of the effect under investigation.

## Conclusion

In the present paper, we investigated orthographic neighbourhood size effects in Chinese character recognition using components across multiple levels of the character hierarchy. At the radical level, we defined two neighbourhood size measures based on shared phonetic components (N-pc) and semantic components (N-sc). We found no effect of either. At the level of stroke pattern, we linearised character composition by representing each character using Ideographic Description Sequences, which encode both stroke patterns and their spatial relations. Using this fine-grained representation, we defined N-ch as the number of characters that differ from a target by one substitution of an IDS component. N-ch showed a slightly inhibitory effect, suggesting mild competition from highly similar characters during recognition. However, all these three substitution-based measures share a constraint: they reduce orthographic similarity to a binary assessment, unable to reflect continuous, graded perceptual similarity (Wang and Keuleers, 2024). To address this, we introduced two density measures, WLD-10 (average edit distance to the ten nearest neighbours) and its normalised version, WND-10 (accounting for sequence length). Both measures showed facilitatory effects of orthographic neighbourhood density: characters with denser neighbourhoods (low WLD-10 / lower WND-10) were recognised faster. This suggests that in a hypothetical orthographic space, a character that is closer to other characters can benefit from such orthographic density, possibly at the early stage of word processing. Together with N-ch, they suggest a *mixed* pattern of neighbourhood effects, consistent with what was observed in English (Yarkoni et al., 2008). The present work highlights 1) that selecting appropriate sub-character components is crucial for measuring orthographic similarity and 2) that the hierarchical nature of characters should be recognised in Chinese visual word recognition research. For future research, we recommend controlling for N-ch (neighbourhood size) and WND-10 (normalised neighbourhood density) in studies where co-activation of visually similar characters is a concern, such as in naming or priming paradigms.

## Data Availability

The code and data for computing orthographic neighbourhood size measures and running the statistical analyses are are made available on Github. Researchers interested in neighbourhood size in character recognition can test all measures. They can also, with ease, design their own methods making use of the materials provided.

## Footnotes

The original Chinese definition of stroke pattern reads: “汉字部件是由笔画组成的具有组配汉字功能的构字单位, 简称部件” (National Language Commission, 2009).

Both studies used the term combinability instead of neighbourhood size.

CJKV stands for Chinese, Japanese, Korean, and Vietnamese.

We replace ‘ideographic description character’ with ‘layout descriptor’ to make it explicit.

https://xh.5156edu.com.

摒 *bing3* ‘to exclude’. 拼 *pin1* ‘to piece together’. 杏 xing4 ‘apricot’. 呆 *dai1* ‘dull’. 刘 *liu2* ‘one common surname’. 浏 *liu2* ‘clear’.

To further clarify the effect of WLD-10, we wanted to separate the effects of stroke patterns from those of layout descriptors. Hence, we computed two additional measures. The first is C-10, which was computed by representing characters solely by sequences of stroke patterns. For example, 英 was encoded as 艹央 and 嚻 as 口口頁口口. C-10 had a strong correlation with WLD-10 (r = .68) The second was L-10, computed by representing characters solely by their layout descriptors. For example, 英 was encoded as ⿱ and 嚻 as ⿲⿱⿱. L-10 showed moderate correlation with WLD-10 (r = .48). Data transformation and model selection followed those established in Study 1. Like WLD-10, C-10 and L-10 also showed significant inhibitory effects: a character with neighbours that look more alike is easier to recognise than a character that looks distinct, both in terms of shared stroke patterns (C-10), and shared subcomponent layouts (L-10). Note that L-10 and C-10 use the same operation costs as WLD-10 (addition and deletion = 1; substitution = 2). The results for the analysis using an identical cost of 1 for all operations show a similar pattern. The finding that these different neighbourhood density measures all show inhibitory effects, suggest that character recognition in Chinese not only involves information about the character components but also the abstract spatial relationships that govern the composition of characters. However, the unique variance explained by these neighbourhood density measures, like WLD-10, was next to zero.

## Appendix

### Study 1

For the possible collinearity in Study 1, we followed Hendrix and Sun (2021) and applied PCA with varimax rotation to the predictors in Study 1. Results are presented in Table 12. The second (RC2), third (RC3), and fifth (RC5) principal components were represented by NStrokes, N-sc, and character frequency with exclusively high loadings. Therefore, these RCs can be readily interpreted as measuring the corresponding variables. RC4 was represented mainly by regularity but also showed a moderate loading from consistency. This implies that higher RC4 scores tended to also have higher levels of both regularity and consistency. RC1 (the first component) was primarily associated with consistency with a negative loading from N-pc. This indicates that RC1 contrasted characters with higher consistency and smaller neighbourhood size against those with lower consistency and larger neighbourhood size. Similarly, RC6 (the sixth component) was represented by N-pc but consistency had a loading of –.30. This implies that RC6 contrasted characters with larger neighbourhood sizes and lower consistency against those with smaller neighbourhood sizes and higher consistency (for more information on the interpretation of principal components, see Jolliffe, 2002, Chapter 4).

**Table 12:** Loadings from principal component analysis for predictors used in Study 1.

VARIABLE	RC1	RC2	RC3	RC4	RC5	RC6

NStrokes	.07	.99	–.07	.01	–.11	–.07

character frequency	–.04	–.11	–.01	–.03	.99	.02

N-sc	.05	–.07	.99	.03	–.01	–.06

N-pc	–.24	–.07	–.06	–.04	.02	.96

regularity	.24	.01	.03	.97	–.03	–.04

consistency	.90	.08	.07	.30	–.05	–.30

To explore these components further, we fitted a LMM using the RCs with the model selection unchanged. Results from the PCA-based LMM model in Table 13 indicate that character frequency had a significant facilitatory effect, while NStrokes had a significant inhibitory effect. Although models with raw predictors showed no effect for log N-pc and consistency, the PCA-based model revealed a subtle nuance. RC1, which represented N-pc with moderate negative loadings on consistency, showed facilitatory effect. **When a character has a large neighbourhood in relation to its consistency, it is easier to recognise**. Conversely, RC6, which measured consistency with moderate negative loadings on N-pc, showed inhibitory effect. **When a character has higher consistency relative to its neighbourhood size (N-pc), it takes longer to recognise it**. However, the effect sizes are very small for both RCs, suggesting limited significance in practice. By contrast, the effect of N-sc did not reach significance in the PCA-based model. This likely indicates that PCA reduced correlations among predictors and, in doing so, mitigated a potential suppression effect of N-sc (Friedman and Wall, 2005), which was itself minimal in the original model.

**Table 13:** Results of the fixed effects factors from the LMM with rotated components from PCA in Study 1 (*N* = 2,767). For each rotated component, the primary representative variable is shown. Variables with moderate contributions to the components are listed in descending order of absolute loading values with + showing positive loading and - negative loading.

VARIABLE	PCA-BASED MODEL				

	ESTIMATE	STD. ERROR	*t*	*df*	*p*

Intercept	–1.497	0.023	28.320	–64.170	< .001***

RC1 (N-pc - Con.)	–0.006	0.002	2711.766	–2.874	< .01**

RC2 (NStrokes)	0.046	0.002	2740.083	23.479	< .001***

RC3 (N-sc)	0.002	0.002	2715.640	1.080	.279

RC4 (regularity + Con.)	0.003	0.002	2720.881	1.512	.131

RC5 (character frequency)	–0.090	0.002	2752.641	–45.587	< .001***

RC6 (Con. - N-pc + regularity)	0.009	0.002	2716.008	4.415	< .001***

### Study 2

To ensure direct comparability between the radical-based neighbourhood measures in Study 1 and IDS-based measures in Study 2, we fitted three LMMs for the IDS-based measures using the identical dataset and model setup as in Study 1. Table 14 shows that all the IDS-based measures were statistically significant, consistent with the results reported in Study 2.

**Table 14:** Results for fixed effects factors across the LMMs combining variables from Study 1 and Study 2 (initial *N* = 2,767 characters, 70,534 trials). After identical outlier removal, final trial counts were 69,374 for the N-ch model, 69,370 for the WLD-10 model, and 69,372 for the WND-10 model. The unrounded estimate for N-ch is 0.0004 (SE = 0.0001).

MODEL	VARIABLE	ESTIMATE	STD. ERROR	*t*	*df*	*p*

N-ch Model	(Intercept)	–1.288	0.027	–48.308	48.200	<.001***

	Character frequency	–0.087	0.002	–42.533	2754.000	<.001***

	NStrokes	0.012	0.001	18.221	2743.000	<.001***

	consistency	0.015	0.007	2.066	2719.000	.039*

	regularity	–0.002	0.005	–0.513	2725.000	.608

	N-ch	0.000	0.000	3.390	2727.000	<.001***

WLD-10 Model	(Intercept)	–1.285	0.027	–48.386	47.663	<.001***

	Character frequency	–0.087	0.002	–42.555	2753.924	<.001***

	NStrokes	0.011	0.001	15.873	2739.439	<.001***

	consistency	0.012	0.007	1.546	2719.351	.122

	regularity	–0.002	0.005	–0.422	2725.011	.673

	WLD-10	0.007	0.002	3.377	2738.607	<.001***

WND-10 Model	(Intercept)	–1.297	0.027	–47.720	52.251	<.001***

	Character frequency	–0.087	0.002	–42.642	2754.705	<.001***

	NStrokes	0.012	0.001	18.316	2746.701	<.001***

	consistency	0.012	0.007	1.557	2721.490	.120

	regularity	–0.002	0.005	–0.458	2725.904	.647

	WND-10	0.094	0.028	3.402	2724.459	<.001***
